# Supplementary material for: Dermal appendage-dependent patterning of zebrafish atoh1a+ Merkel cells
Source: eLife. 2023 Jan 17;12:e85800. doi: 10.7554/eLife.85800 (PMC9901935; doi:10.7554/eLife.85800)
Supplement: Figure 2—source code 1. [file elife-85800-fig2-code1.zip › 23-12-2022-RA-RC-eLife-85800/Figure 2Source Code 1.docx]

// this ImageJ macro was used to assist in quantification of axon association with Merkel cells in the zebrafish epidermis

// this macro assumes you have previously run 3d object counter and saved that data

//open image

path = File.openDialog("Select image file");

open(path); // open the file

path = File.openDialog("Select statistics file");

open(path); // open the file

//retrieves scale from metadata and assigns pixel width and height based on this information

getPixelSize(unit, pw, ph);

//this gets the total number of cells generated from 3d object counter

s = getValue("results.count");

print(s);

//for loop to itterate through the objects from the results table

for (i=0; i<s; i=i+1) {

print(i);

//section 1

//section 1 plots a circle around the cell you are analyzing so you know what cell to measure

//makeOval originally plots the circle with the corner at the coordinates so these factors are used to adjust it

//to plot the circle centered at the center of mass x and y coordinates

c = (3.5/pw);

cc = (7/pw);

//gets x and y coordinates and adjusts them

xcord1 = (getResult("X", i)-c);

ycord1 = (getResult("Y", i)-c);

Stack.setDisplayMode("composite"); //makes a composite image

Stack.setActiveChannels("110"); //turns on the channel used for cell diameter annotation

//plots the oval

makeOval(xcord1, ycord1, cc, cc);

//section 2

//section 2 is where you measure the cell diameter and a circle gets plotted around your cell

//a specific factor larger than the diameter so you can assess what is within that distance away from your cell

//prompts you to use the line tool to annotate the cell diameter

waitForUser("make diameter roi");

//adds your diameter line to the roiManager and measures the length

roiManager("add");

roiManager("select", 0);

roiManager("Measure");

//multiplies the diameter from the above step by a factor or 1.1

D = (1.1 * getResult("Length"));

close("Results");

close("ROI Manager");

print(D);

//section 3

//section 3 plots a circle centered on your center of mass coordinates that is a factor of 1.1 wider than the annotated diameter

//and runs a 3d projection of the square bounding that circle

//factors for adjusting to plot the circle diameter D centered at center of mass x and y coordinates

f = ((D/2)/pw);

ht = (D/pw);

//open statistic file from running 3d object counter

waitForUser("click on statistics file");

//gets x and y coordinates and adjusts them

xcord = (getResult("X", i)-f);

ycord = (getResult("Y", i)-f);

//plotting new circle

makeOval(xcord, ycord, ht, ht);

//3d projecting and increasing the size for viewing

run("3D Project...");

run("In [+]");

run("In [+]");

run("In [+]");

run("In [+]");

run("In [+]");

run("In [+]");

Stack.setDisplayMode("composite"); //makes a composite image

Stack.setActiveChannels("110"); //turns on the channel used for cell diameter annotation

waitForUser("click full image"); //need to click original image before proceeding to next cell

}
